# Dr. Stokes on a Poisonous Root among Gentian

**Published:** 1813-02

**Authors:** Jonathan Stokes

**Affiliations:** Chesterfield


*To the Editors of the Medical and Physical Journal.*

GENTLEMEN,

I AM sorry to announce to the medical world, and to the public brewers, the re-appearance of a poisonous root among gentian, from which it differs, in being whitish-brown, brittle, and insipid. It requires however good day-light, and an attentive eye, to separate it. I recommend the druggist not to select the extraneous roots, but to pick out the gentian, cutting across and tasting those pieces which appear at all ambiguous. I would advise the adventitious root not to be thrown away, as those who may have been in the habit of using aconite may wish to give it a trial, in those obstinate maladies in which they have been obliged to have recourse either to aconitum neomontanum, or aconitum napellus.

It becomes intermixed with gentian, from that root being dug up early in Spring, when the mountain herbarist has no assistance from leaves and stems to enable him to distinguish one root from another. The only remedy I can suggest is the cultivation of gentian. In this county we cultivate chamomile, elecampane, and valerian ; and I am informed that helleboras niger, which used to be intermixed with poisonous roots, is now raised in Wiltshire. In the woodlands of Derbyshire, and the mountains of Wales and Scotland, gentian and arnica would amply repay the cultivator. Perhaps a gold medal from the Society of Arts would be more efficacious than an edict from the college or a prohibitory clause in an Act of Parliament. I am, Gentlemen,

Your obedient humble Servant,
JONATHAN STOKES.

*Chesterfield, Dec.* 8, 1812.

